# Clinicians' caseload management behaviours as explanatory factors in patients' length of time on caseloads: a predictive multilevel study in paediatric community occupational therapy

**DOI:** 10.1186/1472-6963-10-249

**Published:** 2010-08-23

**Authors:** Niina Kolehmainen, Graeme MacLennan, Jillian J Francis, Edward AS Duncan

**Affiliations:** 1 Health Services Research Unit, University of Aberdeen, Health Sciences Building, Foresterhill, Aberdeen, UK; 2 Aberdeen Health Psychology Group, University of Aberdeen, Health Sciences Building, Foresterhill, Aberdeen, UK; 3 Nursing, Midwifery and Allied Health Professions Research Unit, Iris Murdoch Building, University of Stirling, Stirling, UK

## Abstract

**Background:**

Long waiting times and large caseloads are a challenge to children's therapy services internationally. Research in hospital-based healthcare indicates that waiting times are a function of throughput, and that length of care episode is related to clinicians' caseload management behaviour (i.e. actions at assessment, treatment, post-treatment, and discharge). There have been few attempts to study this in community health services. The present study investigated whether community occupational therapists' behaviour predicts children's length of time (LoT) on caseloads.

**Methods:**

Retrospective survey of case notes of children recently discharged from occupational therapy services. Using cluster random sampling, case notes were drawn from therapy records in six NHSScotland Health Boards. Data about therapists' behaviours of assessing, treating, reviewing and discharging, together with child characteristics, were used to construct regression models of factors related to LoT.

**Results:**

Twenty-six therapists [median(IQR) time in paediatrics 8(6-13) years] and 154 of their cases [mean(SD) age 7(3) years; median(IQR) LoT 10(3-21)] were included. A multi-level model, adjusting for clustering, for therapists' actions of communicating assessment outcomes to parents, providing treatment, and placing the child on review, and for a diagnosis of cerebral palsy, explained 44% of variation in LoT.

**Conclusions:**

Occupational therapists' caseload management behaviours are associated with children's LoT on caseloads. Further research is required to investigate the direction of relationships between therapists' behaviours and LoT; and the relationships between contextual factors, therapists' caseload management behaviours and LoT. Further exploration of therapists' beliefs about caseload management could also be useful in identifying possible factors contributing to variation between therapists.

## Background

Timely and equitable access to treatments is a high priority both to those using, and to those delivering, healthcare services [1]. In children's services both the benefits of early intervention and the possible harms associated with delayed intervention have been widely documented [2-5]. Parents' dissatisfaction with services has been found to be triggered by lack of access to and availability of care rather than clinical outcomes [6]. Yet long waiting times and large caseloads are a challenge to children's therapy services internationally [7-11].

To date, there have been few attempts to systematically study the determinants of waiting times in children's community health services [12], or community health services more broadly [13-16]. Research in hospital-based healthcare indicates that waiting times are a function of throughput [17] (i.e. flow of patients from the start to the end of a care episode), and that throughput is influenced by a range of factors at different levels of care delivery, including (i) *patient charatecteristics *(e.g. age, severity of condition, social circumstances) [18]; (ii) *clinicians' behaviour *(i.e. actions at assessment, treatment, post-treatment, and discharge, and underlying beliefs about these actions and the situation) [18-23]; (iii) *organisational factors *(e.g. patient pathways, service and staff structures, organisational procedures and resources) [17,18,21-24]; and (iv) *system and ecological factors *(e.g. methods for financing health services, location and availability of public transport) [18,25].

Unsurprisingly, variables at these different levels have been found to be interlinked [18]. What is perhaps less obvious is that clinicians' behaviour appears to mediate the effects that patient characteristics and organisational and systems factors have on throughput and the length of care episodes [18-20]. For example, clinicians could be limiting efficient allocation of the resources by keeping clients on caseloads for longer than clinically needed [15,26,27]. This is consistent with evidence indicating that, even after accounting for patient and organisational variables, clinicians' behaviour is a strong determinant of the care provided to patients [28].

Whilst acknowledging the considerable differences in systems and contexts of service provision between hospital and community health care, clinicians' behaviour could be one of the key variables relevant to investigating throughput and length of care episodes in community health care. Specifically, evidence of behaviours predicting children's length of time on caseload could be used to develop a behaviour change intervention to improve clinicians' caseload management.

This study explored the extent to which therapists' caseload management behaviours explain variation in children's length of time (LoT) on caseloads. Specifically, the focus was on children's occupational therapists in Scotland as the caseload management problems in these services are considerable [9]. Specifically, this study investigated whether, after adjusting for children's characteristics, therapists' behaviours predict children's LoT on caseloads. The related objectives were (i) to describe therapists' current caseload management behaviours (both sequence and frequency of the behaviours); (ii) to identify the variables that explain variation in LoT; and (iii) to establish the magnitude of the relationships between these variables and LoT.

## Methods

A retrospective design was used. The dependent variable was clients' LoT on caseload, operationalised as time in months from first appointment to date of discharge. Independent variables were child characteristics (age in months at first appointment, gender, and medical diagnoses listed in Table 1) and therapists' caseload management behaviours (listed in Table 2). The specific caseload management behaviours were selected from a review of occupational therapy textbooks [29,30] and the authors' (NK and ED) experience of occupational therapy practice. These chosen behaviours mirror those included in professional standards for occupational therapists caseload management [31] (published after the conduct of the present study). Services within which the therapists worked were described by their size (small was < 10 therapists, mid-size was 10-20 therapists, large was > 20 therapists) and geographical attributes (urban, sub-urban, rural).

**Table 1:** Codes used to record children's diagnoses from case notes

1	Developmental coordination disorder/dyspraxia/general developmental delay
2	Attention disorders/Autistic spectrum disorder/Asperger's Syndrome/Tourette's Syndrome
3	Cerebral palsy
4	Other (e.g. spina bifida/muscular dystrophy/juvenile idiopathic arthritis)
5	No medical diagnosis

**Table 2:** Response rates and sample sizes for the six participating Health Boards

NHS Boards	Response rate for therapists (n/N^†^)	Number of therapists included in the final sample	Number of case notes included in the final sample
A: Large, urban & suburban	69% (9/13)	6	32

B: Mid-size, suburban	66% (6/9)	6	39

C: Mid-size, suburban	44% (4/9)	4	21

D: Mid-size, urban & rural	33% (3/9)	3	23

E: Small, urban	100% (5/5)	4	20

F: Small, rural	80% (4/5)	3	19

**Total (Range):**	**33-100%**	**26**	**154**

^† ^N is the number of therapists sampled from the service

The study was part of a wider programme of research concerning children's occupational therapists' caseload management in the UK. It was approved by the North of Scotland Research Ethics Committee 1 (ref: 07/S0801/55).

### Sampling

A computer-generated random number table was used to sample six NHSScotland (i.e. National Health Service) mainland occupational therapy services from a list of Scottish children's occupational therapy managers. The sampled managers were contacted and with their agreement all six services were included in the project.

Occupational therapists were randomly sampled from each service: 5, 9 and 13 therapists from small, mid-size and large services, respectively. Eligibility criteria for therapists were: employed by the participating service; had a clinical involvement for a minimum of two days a week with children living at home; and had a minimum of two cases discharged within the past five months. Therapy assistants and technical instructors were excluded as their responsibility over clients' caseload management is limited.

From each participating therapist, eight discharged cases were sampled using a random number table. Where therapists had fewer than eight discharged cases, all discharged cases were included. Parents of the children whose case notes had been sampled were informed about the study and provided an opportunity to opt out. The eligibility criteria for children were that they had been previously seen by a participating occupational therapist and had been discharged a maximum of five months before sampling. Children who had been allocated to a therapist but had not attended any appointments were excluded.

### Materials and procedures

The data were collected from children's case notes. The first author extracted the LoT data, child characteristics and therapists' behaviours by using a structured form, with guidance notes for classifying data and a policy for coding ambiguous data (available from the authors). Dates (year and month) for calculating both LoT and child's age were recorded as presented in case notes. The presence of any of the diagnoses and the caseload management behaviours were recorded. Diagnoses were recorded as categories, as opposed to verbatim, to ensure anonymity of the data [32].

The data were collected September 2007 - January 2008; were initially manually recorded in the software package Microsoft Access, and then transferred to statistical packages. The data relating to therapists' current grade and number of years in professional practice were collected as part of the wider research programme [33] and entered in Microsoft Excel.

### Sample size

Due to a lack of previous studies in the field, no data were available for a formal power calculation. However, we initially aimed for 15 therapists and 150 cases to allow estimation of each therapist behaviour univariately in a simple regression model (adjusting for child level factors) and identify candidate behaviours that predicted LoT. Fifteen therapists gives an effective sample size of 15 at the therapist level (i.e. assuming the worst case scenario, i.e. that there is no variation between cases within therapists, there would be 15 data points) and allowed for the modelling of two to three therapist behaviours in a multiple regression model. Based on other studies with professionals [34], a response rate of 50-60% was expected.

### Data Analysis

The data were initially described using mean(SD) (for age), median(IQR, interquartile range) (for LoT), or proportions (for gender, diagnoses and the caseload management behaviours). Multilevel (ML) linear regression models, adjusted for clustering at the level of occupational therapist [35], were used to explore the variation in LoT. Variation in LoT due to child characteristics were initially explored using univariate linear regression. Similarly the relationships between LoT and selected caseload management behaviours were explored univariately. Variables significant at the p = 0.05 level were then combined in a ML multiple linear regression model. Due to the positively skewed distribution of LoT robust standard errors were estimated [36] to ameliorate potentially spuriously narrow confidence intervals and low p-values [37]. Interactions between selected caseload management behaviours were explored by entering an interaction term in the final model. All estimates are presented with 95% confidence intervals. Descriptive analyses were performed in SPSS [38] and ML modelling was performed in Stata [39] using *xtreg *and *robust *commands.

## Results

Data of included participants at each level of the random cluster sampling (service, therapist, case notes) are presented in Table 2. Participating services included a range of community occupational therapy services across NHSScotland, varying both in their size and geographical location. The response rate for therapists within the services ranged from 33-100%. Thirty-one therapists responded (62%); five of them did not meet the inclusion criteria (three did not have at least two recently discharged cases and two did not have a minimum of two days per week clinical involvement with children living at home) resulting in a final study population of 26 (52%) therapists. Reflective of the occupational therapy population [9,40], majority of the included therapists were female (96%) and at Senior level (93%). Two therapists (7%) were managers. Median for time in paediatric practice for the participants was 8 years (IQR = 6-13), which is similar to that observed in other studies of children's occupational therapists in the UK [41]. Median for time as a qualified therapist was 12 years (IQR = 9-20).

The number of cases sampled per therapist ranged from 2-8, totalling in 154 cases. Fourteen (54%) of the participating therapists had discharged fewer than eight cases within the past five months.

Mean age for the included cases was 7 years (SD = 3) corresponding to the typical age range of children seen by occupational therapists [41]. The spread of diagnoses was similar to that observed in other studies of children's occupational therapy in the UK [41]. The three most common diagnostic groups were: cerebral palsy (CP) (8%); attention difficulties, Autistic Spectrum Disorder, Asperger's Syndrome or Tourette's (17%); and developmental coordination disorder, dyspraxia or general developmental delay (DCD/GlobalDelay) (14%). Over a third (33%) of the cases did not have a diagnosis. Twelve percent (12%) had more than one diagnosis.

Median LoT for all cases was 10 months (IQR = 3-21). Three child characteristics explained a significant amount of variation in LoT on caseload: age (in months), diagnosis of CP, and diagnosis of DCD/GlobalDelay. On average, older children remained on caseloads for shorter time [-0.1 months, 95% confidence interval (CI) -0.2 to -0.02; p = 0.02]. For example, an increase in age by one year was associated with a reduction of 1.2 months in time on a caseload (95% CI 0.3 to 2.6, p = 0.02). When compared to children without a diagnosis, children with DCD/GlobalDelay were likely to remain on caseloads for 15 months (95% CI 5 to 24; p < 0.01) longer, and children with CP for 44 months (95% CI 31 to 59; p < 0.01) longer. The total amount of variation in LoT explained by these three variables was 29%.

### Length of time on caseloads and therapists' behaviours

From the case note data, therapists performed assessment behaviours with nearly all children, after which some children were provided with treatment, some were placed on review, and some were discharged. Of the children who were provided with treatment or placed on review, only a limited proportion had therapy goals and plans. After receiving treatment or being reviewed, some children were discharged immediately, whilst others moved from treatment to review or vice versa before being discharged.

Frequencies with which therapists performed the investigated behaviours are reported in Table 3. There was little variation in therapists' assessment and discharging behaviours, and only some variation in forming therapy goals and plans, making these unlikely behaviours to explain variation in LoT. There was considerable variation in providing treatment, including some variation in the number of treatment sessions provided [median(IQR) 7(4-12)] but limited variation in the frequency with which sessions were provided (67% received weekly sessions). There was considerable variation in placing children on review, but only some variation in evaluating progress towards therapy goals and reporting treatment outcomes. The relationships between LoT and ten behaviours (Table 4) were further investigated univariately. Four of these behaviours explained significant variation (p < 0.05) in LoT. These were: communicating assessment outcomes to parents in writing, providing treatment, placing the child on review and circulating a discharge summary or letter to parents.

**Table 3:** Presence of the different caseload management behaviours in the included case notes (n = 154)

		Proportion of cases in which the behaviour was recorded
	**Assessment:**	
1.	Established child's strengths	93%
2.	Established child's difficulties	97%
3.	Communicated assessment outcomes to parents/carers in writing	83%

	**Formulating therapy goals and plans**	
4.	Established treatment goals	33%
5.	Established methods for achieving treatment goals	26%

	**Treatment:**	
6.	Provided treatment	57%

	**Evaluating progress:**	
7.	Monitored progress against treatment goals	19%
8.	Reported (i.e. established and recorded) treatment outcomes	26%
9.	Placed the child on review	47%
10.	Established a goal for placing the child on review	12%

	**Discharging:**	
11.	Established reasons for discharge	72%
12.	Provided a discharge summary or letter	85%
13.	On the summary or letter, stated criteria for a re-referral to the service	5%
14.	Circulated the summary or letter to parents/carers	79%

**Table 4:** Behaviours explored univariately in relation to length of time (LoT) on caseload

Behaviour	Coefficient (months)	p-value	95% Confidence Interval (months)	
*Communicated assessment outcomes to parents/carers in writing*	*-28*	< 0.01**	*-49*	*-8*

Established treatment goals	4	0.37	-5	13

Established methods for achieving treatment goals	-5	0.13	-12	2

*Provided treatment*	*19*	< 0.01**	*11*	*27*

Monitored progress against treatment goals	2	0.74	-8	11

Reported (i.e. established and recorded) treatment outcomes	3	0.78	-5	12
*Placed the child on review*	*27*	< 0.01**	*18*	*35*

Established reasons for discharge	-3	0.70	-16	11

Provided a discharge summary or letter	-22	0.05	-45	0

*Circulated the summary or letter to parents/carers*	*-19*	0.03*	*-36*	*-2*

### Length of time on caseloads and child characteristics and therapists' behaviours

The four behaviours identified in Table 4 were entered in a regression model, together with (1) *age*, (2) *DCD/GlobalDelay*, and (3) *CP*. The model was adjusted for clustering within therapist. *Age*, *DCD/GlobalDelay *and *circulating a discharge summary to parents *no longer explained a significant amount of variation. These were subsequently removed from the model. The four remaining variables of *CP*, *communicating assessment outcomes to parents in writing*, *providing treatment *and *placing on review *explained 44% of variation in LoT. Communicating assessment outcomes in writing was associated with reduction in LoT of 15 months (95% CI -29 to -1; p = 0.04). Providing treatment was associated with an increase in LoT of 7 months (95% CI 2 to 13; p < 0.01), and placing on review was associated with an increase of 20 months (95% CI 13 to 26; p < 0.01). Cerebral palsy (CP) continued to be associated with a considerable increase in LoT [36 months (95% CI 9 to 63; p < 0.01)]. Early exploration of the data had suggested a possible interaction between providing treatment and placing children on review, however, this was not statistically significant [5 months (95% CI -8 to 18; p = 0.44)].

**Figure 1:**
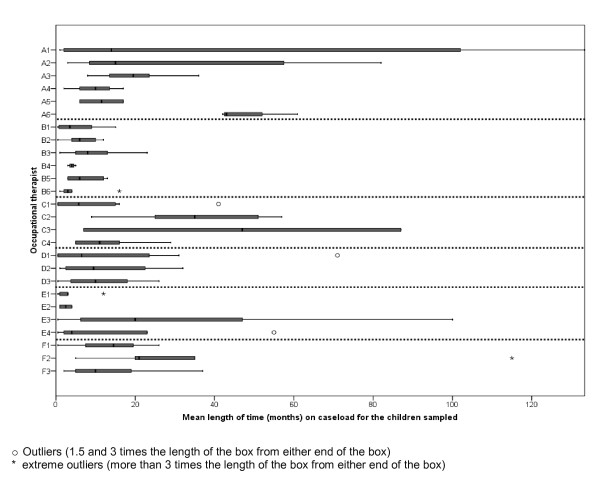
**Boxplots of length of time**. Boxplots of length of time (LoT) for therapists (n = 26) clustered within the six services (from A to F).

Further breakdown of mean LoTs and distribution of the factors related to LoT, according to individual therapist and as clustered within services, is presented in Figure 1 and Table 5. In services B, D and E, mean LoTs for therapists were below or at the sample average, and variation in mean LoTs between therapists was small. In all other services, there were no observed service-level patterns of therapists' mean LoTs.

**Table 5:** Proportion of children with cerebral palsy, therapists' caseload management behaviours, and mean LoT for each therapist within the six services

Service code: therapist	% of cases with cerebral palsy (n/N)^ξ^	Communicated assessment findings to parents in writing (%)^¥^	Provided treatment (%)^¥^	Placed on review (%)^¥^	Length of Time on caseload [Median (IQR)]^±^	
A:1	**50 (4/8)**	75	**50**	50	14	(2-135)
A:2	**13 (1/8)**	75	**50**	**38**	15	(8-70)
A:3	0 (0/8)	**100**	88	50	20	(12-24)
A:4	0 (0/3)	33	100	**33**	10	(2-17)
A:5	**50 (1/2)**	**100**	100	**0**	12	(6-17)
A:6	**33 (1/3)**	0	100	100	43	(42-61)

B:1	0 (0/8)	**100**	63	**25**	**4**	**(1-11)**
B:2	0 (0/8)	**100**	**50**	**25**	**6**	**(4-11)**
B:3	**13 (1/8)**	88	88	50	**8**	**(4-14)**
B:4	0 (0/3)	**100**	**33**	**0**	**4**	**(3-5)**
B:5	0 (0/6)	**100**	**50**	50	**6**	**(3-13)**
B:6	**17 (1/6)**	84	**17**	100	**3**	**(2-7)**

C:1	0 (0/8)	88	**25**	50	**6**	**(1-16)**
C:2	0 (0/6)	84	83	67	35	(21-52)
C:3	**50 (1/2)**	**100**	100	100	47	(7-87)
C:4	0 (0/5)	60	**40**	80	11	(5-23)

D:1	0 (0/8)	88	**38**	50	**7**	**(1-27)**
D:2	0 (0/8)	75	**38**	63	10	(2-24)
D:3	**14 (1/7)**	**100**	57	71	10	(1-26)

E:1	0 (0/5)	**100**	**20**	**20**	**3**	**(1-8)**
E:2	**50 (1/2)**	**100**	100	**0**	**3**	**(1-4)**
E:3	0 (0/8)	**100**	63	50	20	(3-56)
E:4	**20 (1/5)**	40	**40**	**20**	**4**	**(1-39)**

F:1	0 (0/8)	**100**	**50**	75	15	(7-20)
F:2	**40 (2/5)**	60	60	80	21	(21-75)
F:3	0 (0/6)	50	83	50	10	(4-24)

**Total sample:**	**10 (15/154)**	**83**	**57**	**47**	**10**	**(3-21)**

**^ξ ^**In this column, samples consisting children with cerebral palsy are highlighted **bold**

**^¥ ^**In this column, bold is used to indicate where assessment findings were communicated more frequently in writing/treatment was provided less frequently/children were placed on a review less frequently than the sample average

**^± ^**Median LoT shorter than the sample average

In terms of distribution of the factors related to LoT (Table 5), therapists treating children with CP were sampled from all services. There appeared to be a trend for therapists in services B and E to be more consistent in communicating assessment findings to parents in writing, providing less treatment and placing fewer children on review than the sample average. With the exception of one therapist (E3), mean LoTs for therapists in services B and E were below the sample average. For all other services, the frequency of performing the three behaviours and LoTs appeared to vary as much between therapists as between services.

## Discussion

After controlling for child-related factors, individual therapists' caseload management behaviours were associated with variation in the length of time (LoT) that the children remained on therapists' caseloads. From the investigated child characteristics, only a diagnosis of cerebral palsy explained a significant proportion of variation in LoT after adjustments for therapist effects. The other variables included in the final model were: communicating assessment outcomes to parents in writing; providing treatment; and placing children on review.

This study has four major limitations. First, diagnosis was the only measure of the cases' clinical features. It is possible that case characteristics other than diagnosis (e.g. performance in selected activities [42,43]) would better capture the key clinical features that influence therapists' caseload management behaviours. However, there is currently no agreement about what these characteristics are and there are few standardised measures to assess them, leaving diagnosis as the most feasible and reliable variable on which to base this analysis. Second, the study used retrospective data and it is not possible to make conclusions about the directions of potential causal relationships. A further experimental study is required to establish these. Third, the generalisability of the results is limited to children's senior occupational therapists in NHSScotland, as the contextual factors (e.g. policies, models of practice) may be different in other locations and with other therapist populations in a way not considered in this study. The study could be replicated in other settings to further investigate the influence of organisational and system-level factors on therapists' caseload management behaviours, and on the relationship between the behaviours and LoT. Fourth, the therapists sampled from each service may not be fully representative of that service, and the response rate varied between services. It is therefore possible that in reality, there is more or less variation between therapists in specific services than is evident from these data.

From the four variables identified to relate to LoT in this study, therapists' behaviours may be modifiable whereas a diagnosis of cerebral palsy is not. The magnitude of the relationships between length of time on caseload and the identified caseload management behaviours suggests that, if the relationships are causal, changing the behaviours could result in a change in LoT.

### Therapists' behaviours and length of time on caseload

In terms of developing hypotheses about the directions of the relationships identified in this study, providing treatment typically takes place early in the care process and inevitably means that the therapist has to keep the child on caseload. This suggests that the direction of this relationship could be from treatment provision to longer length of time on caseloads. Communicating assessment findings to parents also takes place early in the process; however, the relationship between this and LoT is more difficult to theorise. This apparent relationship could result from therapists discharging the child and providing a written assessment report as a discharge summary, or it could be that communicating assessment findings in writing is influenced by a confounding variable related to it and LoT (e.g. therapists' approach to communicating with parents). Placing a child on review typically takes place after the child has stayed on the caseload for some time, and thus it is difficult to draw further conclusions about the direction of this relationship. It is possible that children are placed on review and subsequently remain on caseloads for longer, or that children are reviewed because they have remained on caseloads. An experimental study is required to further investigate the nature and direction of the relationships identified. Further exploration of therapists' beliefs about caseload management could also be useful in identifying other possible factors contributing to variation between therapists.

The behaviours associated with LoT do not occur in isolation but as part of a sequence of caseload management behaviours that may be interdependent. The present study identified that therapists formulate goals, monitor progress towards goals and report outcomes infrequently. Whilst these behaviours were not related to LoT in this study, it may be important to understand the processes between the behaviours. For example, therapy goals can be hypothesised to direct [44] therapists to provide treatment efficiently, and the lack of these goals could contribute to treatment provision for children who may not require treatment. Further exploration of the processes related to therapists' caseload management behaviours is required in order to develop a theoretically coherent understanding of caseload management.

### Diagnosis and contextual factors

The finding that a diagnosis of cerebral palsy explains LoT is unsurprising as these children can be perceived as having 'complex needs'. In terms of the direction of any causal relationship, having cerebral palsy is likely to contribute to longer length of time on caseload, as the opposite (i.e. time on caseload contributing to cerebral palsy) is unlikely. However, the substantially long times that these children are kept on caseloads is problematic in that there is currently scarce evidence about the benefits of the occupational therapy interventions for them [45]. In the broader context of efficient resource use, it would be important to establish good-quality evidence about the costs and benefits of keeping these children on caseloads for such long periods.

Descriptive exploration of the clustering of the children's mean lengths of time on caseloads and therapists' behaviours within occupational therapy services suggested that organisational and/or contextual factors may have some influence on therapists' behaviours and the lengths of time on caseloads. Further exploration of the relationships between the different caseload management behaviours and the broader context within which they are carried out is required.

### Implications for future research

This study supports the hypothesis of an association between therapists' behaviours and children's length of time on caseload. If future research provides plausible evidence for causal relationship then there is the potential for reducing children's length of time on caseloads by targeting therapists' behaviours. Future research could investigate how treatment provision and placing children on review relate to other caseload management behaviours, that is, what are the processes linking the behaviours and children's length of time on therapists' caseloads. Further research is also required to investigate the relationships and interactions between therapists' behaviours and the context (including the child, the organisation, etc) within which the behaviours are carried out. Finally, further exploration of therapists' beliefs about caseload management could also be useful in identifying other possible factors contributing to variation between therapists.

## Conclusions

Occupational therapists' caseload management behaviours are associated with children's LoT on caseloads. Further research is required to investigate the direction of relationships between therapists' behaviours and LoT; and the relationships between contextual factors, therapists' caseload management behaviours and LoT. Further exploration of therapists' beliefs about caseload management could also be useful in identifying possible factors contributing to variation between therapists.

## Competing interests

The authors declare that they have no competing interests.

## Authors' contributions

All authors contributed to design of the study. NK collected, processed, analysed and interpreted the data with advice from GM and JF. NK drafted the manuscript. GM, JF and ED critically revised the manuscript for important intellectual content. All authors had full access to the data, and saw and approved the final manuscript. NK, GM and JF are guarantors, and take responsibility for the integrity of the data and the accuracy of the data analysis.

## Pre-publication history

The pre-publication history for this paper can be accessed here:

http://www.biomedcentral.com/1472-6963/10/249/prepub
